# Chymase inhibition retards albuminuria in type 2 diabetes

**DOI:** 10.14814/phy2.14302

**Published:** 2019-12-23

**Authors:** Benjamin J. Bivona, Shinji Takai, Dale M. Seth, Ryousuke Satou, Lisa M. Harrison‐Bernard

**Affiliations:** ^1^ Department of Physiology Louisiana State University Health Sciences Center New Orleans LA USA; ^2^ Department of Innovative Medicine Osaka Medical College Takatsuki City Osaka Japan; ^3^ Department of Physiology Tulane University School of Medicine New Orleans LA USA; ^4^ Present address: University of Texas MD Anderson Cancer Center Houston TX USA

**Keywords:** angiotensin II, db/db mouse, fibrosis

## Abstract

Chymase released from mast cells produces pro‐fibrotic, inflammatory, and vasoconstrictor agents. Studies were performed to test the hypothesis that chronic chymase inhibition provides a renal protective effect in type 2 diabetes. Diabetic (*db/db*) and control mice (*db/m*) were chronically infused with a chymase‐specific inhibitor or vehicle for 8 weeks. Baseline urinary albumin excretion (UalbV) averaged 42 ± 3 and 442 ± 32 microg/d in control (*n* = 22) and diabetic mice (*n* = 27), respectively (*p* < .05). After administration of chymase inhibitor to diabetic mice, the change in UalbV was significantly lower (459 ± 57 microg/d) than in vehicle‐treated diabetic mice (645 ± 108 microg/d). U_NGAL_V was not different at baseline between diabetic mice that would receive the chymase inhibitor (349 ± 56 ng/d, *n* = 6) and vehicle (373 ± 99 ng/d, *n* = 6) infusions, but increased significantly only in the vehicle‐treated diabetic mice (*p* < .05). Glomeruli of diabetic kidneys treated chronically with chymase inhibition demonstrated reduced mesangial matrix expansion compared to glomeruli from untreated diabetic mice. Plasma angiotensin II levels were not altered by chymase inhibitor treatment. In summary, chronic chymase inhibition slowed the progression of urinary albumin excretion in diabetic mice. In conclusion, renal chymase may contribute to the progression of albuminuria in type 2 diabetes renal disease.

## Graphical abstract

The most significant finding of the present study is the identification that chronic inhibition of chymase in the db/db type II diabetic mouse results in renoprotection as reflected by a significant attenuation of urinary albumin excretion and mesangial expansion/glomerular injury. These studies suggest that chymase inhibition may represent a novel therapeutic target in cardiovascular and renal disease.

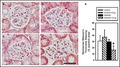

## 1 INTRODUCTION

Diabetes affects 29 million Americans, 390 million people worldwide, and is the 6th leading cause of death in the US. Obesity has been identified as the principal risk factor associated with the rising prevalence of type II diabetes (Ford, Giles, & Dietz, 2002). Diabetic nephropathy is the most common cause of kidney failure in the Western world, and current therapies do not arrest disease progression. This chronic and debilitating disease is characterized by progressive albuminuria, declining glomerular filtration rate, and increased risk for cardiovascular disease, eventually requiring dialysis. Pharmacologic drugs that inhibit the actions of angiotensin‐converting enzyme and the angiotensin type 1 receptor are registered for delaying the onset and slowing the progression of diabetic nephropathy in humans; however, these drugs do not halt disease progression to end‐stage kidney failure. These treatments do not consistently reduce proteinuria, which is not only a powerful predictor, but also a promoter of renal progression. Our overall goal was to identify new therapeutic targets for the prevention, treatment, and reversal of diabetic renal disease. Our recent studies support a role for increased chymase activity in the renal vasculature of type II diabetic db/db mice (Harrison‐Bernard, deGaravilla, & Bivona, 2013; Park et al., 2013) and thus provide a novel translational approach to human disease.

Chymase is the primary angiotensin II (ANG II) forming enzyme in the human heart. Inhibition of chymase has been shown to provide cardioprotection (Ferrario & Mullick, 2017). Chymase inhibition attenuates cardiac fibrosis and dysfunction after myocardial infarction (Kanemitsu et al., 2006), progression to heart failure after autoimmune myocarditis (Palaniyandi et al., 2007), development of abdominal aortic aneurysm (Inoue et al., 2009), and interstitial fibrosis in renal obstruction (Fan et al., 2009) indicating that chymase is critical for fibrosis. The relevance of chymase in cardiovascular disease is being tested in clinical trials by evaluating the effects of the chymase inhibitor BAY1142524 in patients with post‐myocardial infarction left‐ventricular dysfunction (CHIARA MIA 1, CHIARA MIA 2). There are currently no clinical trials evaluating the beneficial effects of chymase inhibition in renal disease.

Increased chymase expression has been observed in humans with diabetic nephropathy (DN) (Huang, Chen, & Truong, 2003; Ritz, 2003), IgA nephropathy (Konishi et al., 2008; Sakamoto‐Ihara et al., 2007), autosomal dominant polycystic kidney disease (McPherson et al., 2004), hypertensive nephropathy (Welker et al., 2008) and obstructive uropathy (Pons et al., 2017) suggesting a central role of chymase in many forms of kidney disease in humans. Increased chymase expression in mesangial and vascular smooth muscle cells in human DN (Huang et al., 2003) suggests that pharmacological blockade of chymase may provide beneficial effects. Hyperglycemia‐induced activation of chymase‐dependent ANG II formation in podocytes promotes progressive podocyte injury and loss in diabetic nephropathy (Durvasula & Shankland, 2008). Recent studies indicate that mMCP‐4 (chymase) deficient mice exhibit lower proteinuria, blood creatinine, blood urea nitrogen levels and less severe renal damage in a model of glomerulonephritis indicating an aggravating role of renal chymase in the disease progression (Scandiuzzi et al., 2010). Similarly, mMCP‐4 (mouse mast cell protease; chymase)‐deficient mice exhibit reduced fibrosis following partial ureteral obstruction (Pons et al., 2017). Chronic inhibition of chymase (Suc‐Val‐Pro‐Phe‐P(OPh)2) reduced urinary albumin excretion and deposition of extracellular matrix components in the kidney of type I diabetic rats (Zhang, Huang, & Bai, 2016). Shi and colleagues have provided key evidence for a critical role of mast cells in the progression of diet‐induced obesity and diabetes in mice (Liu et al. (2009); Wang & Shi, 2011; Xu & Shi, 2012; Zhang & Shi, 2012). Therefore, the concept of chymase‐dependent production of ANG II in pathogenic conditions involving the heart, vasculature, and kidney provide strong support for the prediction that inhibition of this pathway may lead to new effective therapeutic approaches.

The *db/db* mouse exhibits metabolic disturbances of diabetes mellitus similar to the characteristics of humans and thus make this a valuable model of type II diabetic kidney disease (Breyer et al., 2005; Hummel, Dickie, & Coleman, 1966; Sharma, McCue, & Dunn, 2003). Since chymase expression increases in humans with diabetic nephropathy, studies were performed in leptin receptor‐deficient type 2 diabetic mice to test the hypothesis that chronic chymase inhibition reduces albuminuria and glomerular mesangial matrix and fibrosis.

## 2 MATERIALS AND METHODS

### 2.1 Animals

The procedures used in this study were approved by the Animal Care and Use Committee of Louisiana State University Health Sciences Center and conducted according to the *National Institutes of Health Guide for the Care and Use of Laboratory Animals*. Experiments were performed in 6‐week‐male diabetic *db/db* (*n* = 28) (BKS.Cg‐Dock7 < m> +/+ Lepr < db>/J) and control *db/m* (*n* = 22) (BKS.Cg‐Dock7 < m> +/+ Lepr < db>/J) mouse littermates (The Jackson Laboratory, Bar Harbor, Maine). Osmotic minipumps (Alzet Model 1001; 100 μl total volume, 2.6 μl/day infusion rate, 4 week infusion period) were implanted subcutaneously along the lateral abdomen into 6‐week‐old male diabetic and littermate control mice under continuous inhaled isoflurane (2.5%–4%) while on a heating pad. Mice were administered pre‐operative antibiotics (penicillin; 0.025 ml; 150,000 units/ml) intramuscularly. Minipumps contained a specific inhibitor of the enzyme chymase (Suc‐Val‐Pro‐PheP(OPh)2) delivered at a dose of 1 microgram/g BW/min which has been shown to provide chymase inhibition (Okamoto, Takai, & Miyazaki, 2004; Okamoto, Takai, & Yamada, 2002). Vehicle animals were implanted subcutaneously with an osmotic minipump containing 25% DMSO. Minipumps were removed from mice at the end of the 4 week period under anesthesia and a new minipump was inserted subcutaneously into the same position. Water and food intake, and urine output were measured at time zero, 4 and 8 weeks post infusion on mice housed individually in metabolic cages for 24 hr. Urine samples were analyzed for albumin and neutrophil gelatinase‐associated lipocalin (NGAL) concentrations using a commercially available indirect competitive ELISA (Mouse Urine Albumin ELISA Kit #1011, Exocell/Albuwell M) and direct sandwich ELISA (Lipocalin‐2/NGAL ELISA Kit #DY1857, R&D Systems), respectively, according to the manufacturer's instructions to evaluate the progression of kidney injury. Fasting blood glucose levels were measured at time zero and after 8 weeks of infusion in mice housed in metabolic cages following 6 hr of food removal (7 a.m. to 1 p.m.) according to the standard protocol established by The National Institutes of Health (http://www.mmpc.org) and adapted by the Animal Models of Diabetic Complications Consortium (AMDCC; http://www.amdcc.org). All animals were provided ad libitum access to food and water during the study with the exception of food removal for fasting blood glucose measurements.

### 2.2 Histology

Kidneys were immersion fixed in 10% buffered formalin, processed to paraffin, and sectioned at 5 µm. Sections were stained with Masson's trichrome or periodic acid Schiff (PAS) to examine renal fibrosis and mesangial expansion/glomerular injury, respectively. Staining conditions were identical for all slides for each stain. For quantitative analysis of glomerular staining, 5–8 microscopic fields of renal cortex/tissue section/animal were obtained using X10 objective and identical image exposure camera settings for all tissue sections with the microscopist blinded to the animal groups. Each glomerulus in the low power micrograph was manually traced and the positive PAS (manually selected pink color) or trichrome (manually selected blue color) staining was examined using Image Pro Plus as we have reported previously (Park et al., 2010; Park & Harrison‐Bernard, 2008; Prieto‐Carrasquero et al., 2004). RBCs were not included in the analysis of positive staining. Positive glomerular staining is expressed as a percentage of glomerular area using the Image Pro Plus software. On average, 67 glomeruli were analyzed per animal per stain. Approximately 1,100 total glomeruli were analyzed in a total of 110 microscopic images per stain.

### 2.3 Plasma and tissue ANG II levels

Blood, total kidneys, and heart were harvested from conscious control and diabetic mice following decapitation. Trunk blood was collected into chilled tubes containing EDTA (5 mM), enalaprilat (20 µM), pepstatin A (10 µM), and 1, 10‐phenanthroline (1.25 mM) as we have described previously (Kobori, Harrison‐Bernard, & Navar, 2001a). Plasma was separated and stored at −20°C until assayed. Immediately after removal, kidneys and hearts were weighed and then homogenized in iced cold methanol. ANG II levels were quantitated by RIA as previously reported (Kobori, Harrison‐Bernard, & Navar, 2001b). ANG II ELISA was performed to determine whether the chymase inhibitor or vehicle interfered with the ANG II measurements. Samples containing 90, 22.5, 5.6 pg/ml ANG II alone (detected concentrations 89.1, 19.3, 5.6 pg/ml), or ANG II plus equal amounts of chymase inhibitor (87.9, 19.1, 5.9 pg/ml), or ANG II plus 1% DMSO (87.5, 19.5, 5.9 pg/ml) were tested. There were no effects of the chymase inhibitor or vehicle on the ANG II concentrations.

### 2.4 Data analysis

Statistical analyses (SigmaStat 3.5, Systat Sofware, Inc.) were performed by one‐way or two‐way ANOVA with Bonferroni post hoc test. Unpaired *t*‐test was used as appropriate. *p* < .05 was considered statistically significant. Values are means ± *SE* (*n* = number of mice).

## 3 RESULTS

### 3.1 Diabetic and control animals

**Figure 1:**
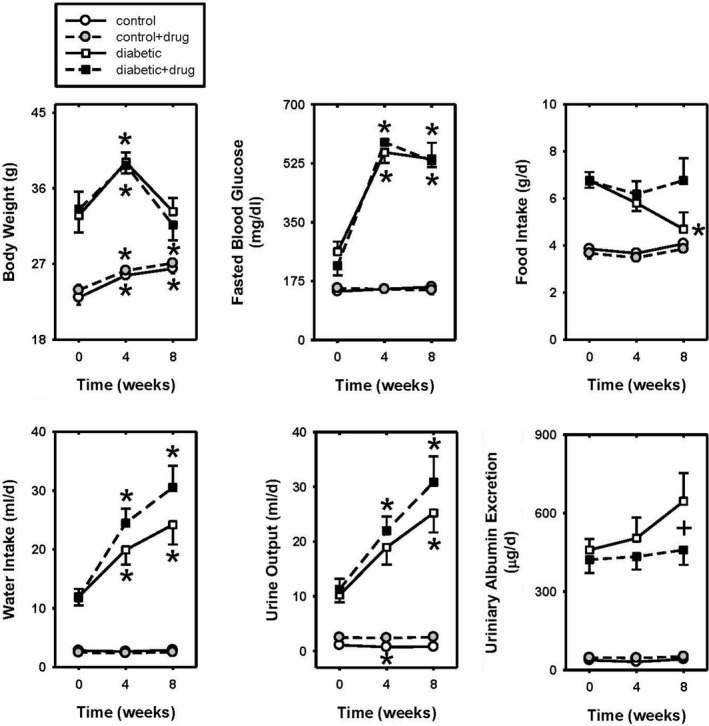
The body weights (a), fasted blood glucose (b), food intake (c), water intake (d), urine output (e), and urinary albumin excretion (f) at time zero and during 4 and 8 weeks of vehicle or chymase inhibitor infusion in control (*n* = 11) and diabetic (*n* = 13) mice. Data are means ± *SEM*; *p < .5 versus time zero of same group; +*p* ≤ .05 vehicle‐treated diabetic versus chymase inhibitor‐treated diabetic

The body weights, fasted blood glucose, food intake, water intake, and urine output were not different between vehicle‐treated and chymase inhibitor‐treated control or diabetic mice during the 8 week study. In diabetic mice during the 8 weeks of infusion, body weights, fasted blood glucose, water intake, urine output, and urinary albumin excretion were significantly greater compared to control mice (Figure 1). There was a significant decrease in food intake in diabetic mice at 8 weeks. The blood glucose, water intake, and urine output levels in the diabetic mice were further elevated at 8 weeks of infusion. Baseline urinary albumin excretion (U_alb_V) averaged 37.5 ± 3 and 47 ± 4 μg/d in control (*n* = 11) and control mice that would receive vehicle or chymase inhibitor treatment (*n* = 11), respectively (Figure 1f). Baseline U_alb_V averaged 459 ± 42 and 422 ± 51 μg/d in diabetic (*n* = 15) and diabetic mice that would receive vehicle or chymase inhibitor treatment (*n* = 12), respectively (Figure 1f; *p* > .05). Baseline U_alb_V for the diabetic mice (442 ± 32 μg/d, *n* = 27) was significantly greater than control mice (42 ± 3 μg/d, *n* = 22). Consistent with our hypothesis, the change in U_alb_V was significantly different between chymase inhibitor (459 ± 57 μg/d) and vehicle (645 ± 108 μg/d) infused diabetic mice after 8 weeks of treatment (Figure 1f). U_alb_V was not altered by chronic chymase inhibition in control mice (Figure 1f). U_NGAL_V was not different at baseline between control mice that would receive the chymase inhibitor (6.3 ± 2.6 ng/d, *n* = 6) and vehicle (3.3 ± 0.8 ng/d, *n* = 6) infusions, respectively. U_NGAL_V was not different at baseline between diabetic mice that would receive the chymase inhibitor (349 ± 56 ng/d, *n* = 6) and vehicle (373 ± 99 ng/d, *n* = 6) infusions, but increased significantly in the vehicle‐treated diabetic mice. U_NGAL_V did not increase during the 8 week infusion with the chymase inhibitor in diabetic or control mice. Baseline U_NGAL_V for the diabetic mice (361 ± 54 ng/d, *n* = 12) was significantly greater than control mice (4.8 ± 1.4 ng/d, *n* = 12). At the time of tissue harvest mice were 14 weeks of age. Diabetic mice exhibited significantly elevated body weight, total kidney weight, and left kidney weights compared to control mice (Table 1).

**Table 1:** Metabolic parameters of control and diabetic mice at tissue harvest

	Control (*n* = 10)	Control + drug (*n* = 9)	Diabetic (*n* = 10)	Diabetic + drug (*n* = 7)
BW *g*	27.0 ± 0.5	26.4 ± 0.7	31.0 ± 1.3*	28.8 ± 1.9
Total kidney wt *mg*	365 ± 14	369 ± 16	451 ± 24*	469 ± 18
Right kidney wt *mg*	176 ± 14	187 ± 16	212 ± 15	222 ± 8
Left kidney wt *mg*	189 ± 3	182 ± 3	236 ± 13*	247 ± 14
Heart wt *mg*	132 ± 4	124 ± 3	122 ± 3	119 ± 3

* *p* < .05 control versus diabetic.

### 3.2 Renal glomerular fibrosis and mesangial expansion

**Figure 2:**
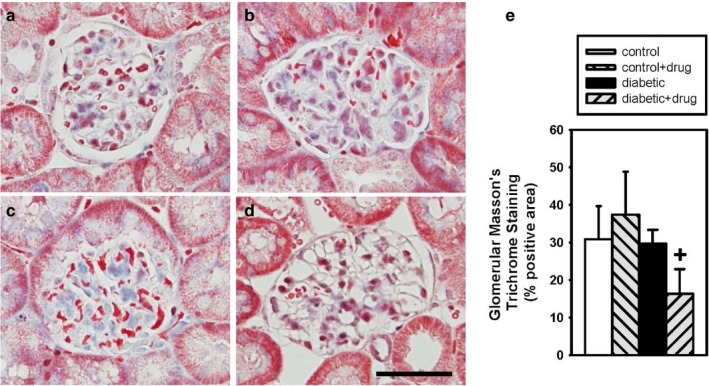
Glomerular Masson's trichrome staining in vehicle‐treated control (□; *n* = 4) (a), vehicle‐treated diabetic (■; *n* = 6) (b), chymase inhibitor‐treated control (gray back hatch; *n* = 3) (c), and chymase inhibitor‐treated diabetic (gray forward hatch; *n* = 4) (d) mice expressed as percent glomerular area staining (e), Data are means ± *SEM*; +*p* ≤ .05 vehicle‐treated diabetic versus chymase inhibitor‐treated diabetic; bar = 50 microns

**Figure 3:**
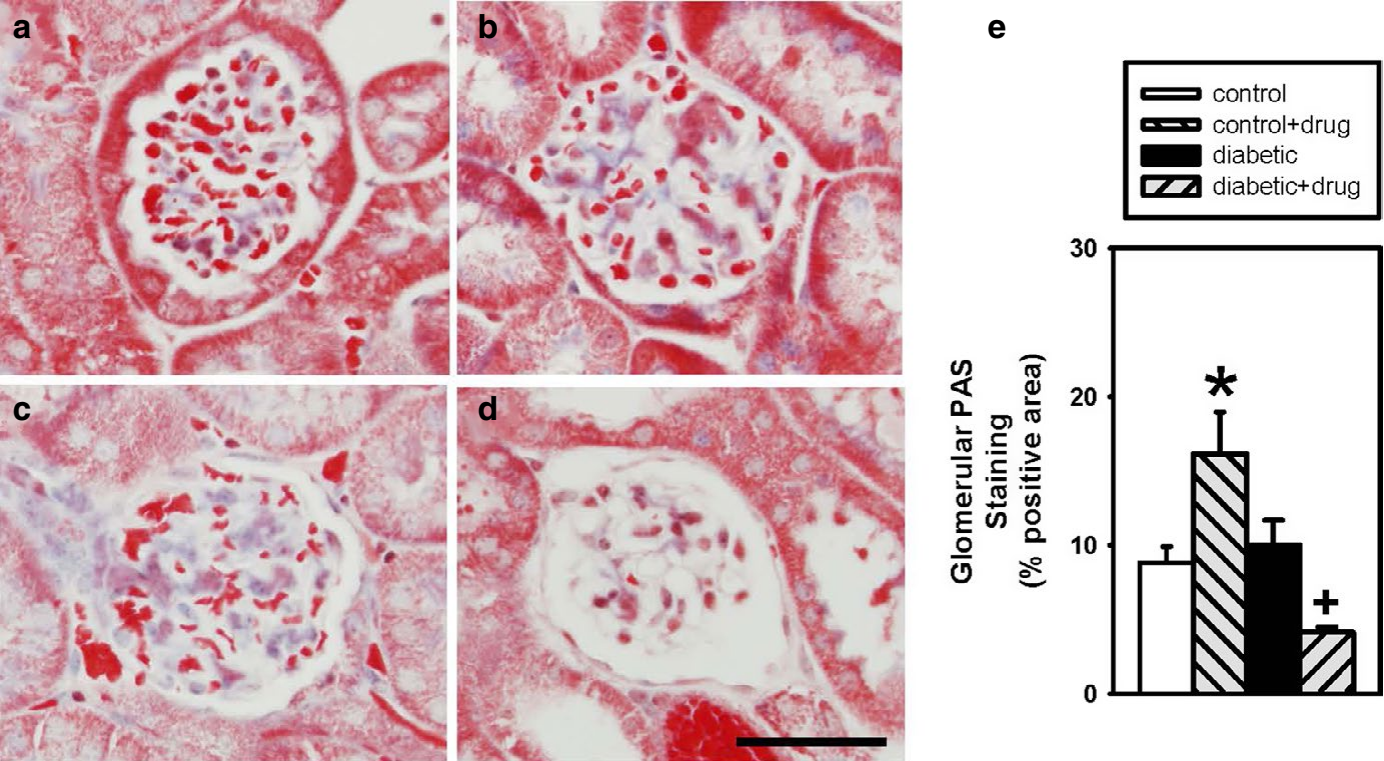
Glomerular periodic acid Schiff (PAS) staining in vehicle‐treated control (□;*n* = 4) (a), vehicle‐treated diabetic (■; *n* = 6) (b), chymase inhibitor‐treated control (gray back hatch; *n* = 3) (c), and chymase inhibitor‐treated diabetic (gray forward hatch; *n* = 4) (d) mice expressed as percent glomerular area staining (e), Data are means ± *SEM*; *p < .05 chymase inhibitor‐treated control versus vehicle‐treated control, +*p* ≤ .05 vehicle‐treated diabetic versus chymase inhibitor‐treated diabetic; bar = 50 microns

Quantitative analysis of the percent positive trichrome staining per glomerular area was not different between vehicle‐treated and chymase inhibitor‐treated (Figure 2c) control mice (Figure 2a and e), while chymase inhibitor‐treated glomeruli (Figure 2d) had a significantly reduced percent positive PAS staining per glomerular area compared to vehicle‐treated (Figure 2b and e) diabetic mice. There was no difference in trichrome staining between vehicle‐treated control and diabetic mice. Quantitative analysis of the percent positive PAS staining per glomerular area was significantly elevated in chymase inhibitor‐treated (Figure 3c) compared to vehicle‐treated (Figure 3a and e) control mice, while chymase inhibitor‐treated glomeruli (Figure 3d) had a significantly reduced percent positive PAS staining per glomerular area compared to vehicle‐treated (Figure 3b) diabetic mice. There was no difference in PAS staining between vehicle‐treated control and diabetic mice.

### 3.3 Plasma and tissue ANG II concentrations

**Figure 4:**
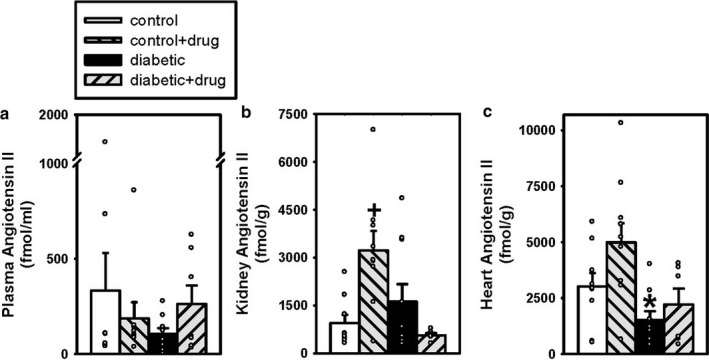
Plasma (a), Kidney (b), and Heart (c) ANG II levels in vehicle‐treated control (□; *n* = 9, 9, 9) and diabetic (■; *n* = 9, 10, 9) mice and chymase inhibitor‐treated control (gray back hatch; *n* = 9, 9, 10) and diabetic (gray forward hatch; *n* = 7, 6, 6) mice. Scatter plot of raw data and bar graphs show mean data ± *SEM*; *p < .05 vehicle‐treated diabetic versus vehicle‐treated control, +*p* < .05 chymase inhibitor‐treated control versus vehicle‐treated control

Plasma ANG II levels were similar in vehicle‐treated and chymase inhibitor‐treated control (332 ± 198 and 187 ± 85 fmol/ml, *n* = 9, 9) and diabetic (106 ± 30 and 262 ± 97 fmol/ml, *n* = 9, 7; *p* = .055) mice (Figure 4a). Kidney ANG II levels were significantly greater in chymase inhibitor‐treated control (3,220 ± 612 fmol/g *n* = 9) compared to vehicle‐treated control (950 ± 255 *n* = 9) mice (Figure 4b). Kidney ANG II levels were not different between vehicle‐treated and chymase inhibitor‐treated diabetic (1617 ± 547 and 563 ± 65 fmol/g *n* = 10, 6; *p* = .08) mice (Figure 4b). Cardiac ANG II levels were significantly lower in vehicle‐treated diabetic (1,525 ± 391 fmol/g *n* = 9) compared to vehicle‐treated control (3,023 ± 602 *n* = 9) mice (Figure 4c). Cardiac ANG II levels were similar in vehicle‐treated and chymase inhibitor‐treated control (3,023 ± 602 and 4,988 ± 861 fmol/ml, *n* = 9, 10) and diabetic (1,525 ± 391 and 2,213 ± 714 fmol/ml, *n* = 9, 6) mice (Figure 4c).

## 4 DISCUSSION

There has been a lack of investigation into the renoprotective effects of chronic chymase inhibition in models of type II diabetes. Our data support the hypothesis that chronic inhibition of chymase in the db/db type II diabetic mouse results in renoprotection as reflected by a significant attenuation of urinary albumin excretion and mesangial expansion/glomerular injury compared to vehicle‐treated diabetic mice. It is possible that the renoprotection is due to reduced barotrauma due to decreases in renal perfusion pressure, reduction in inflammation, and improved podocyte function. These protective effects occurred without a change in fasted blood glucose or plasma, kidney, or heart ANG II concentrations between chymase inhibitor‐treated and vehicle‐treated diabetic mice. However, chymase inhibition produced an increase in kidney ANG II levels and glomerular fibrosis in control mice compared to chymase inhibitor‐treated control mice. This may be a result of locally increased ANG II production that results in elevated pro‐fibrotic factors within the glomerulus. Despite the presence of glomerular fibrosis in chymase inhibitor‐treated control mice, urinary albumin excretion was at normal levels. This finding could raise safety concerns and may limit the clinical use of the chymase inhibitor.

Velez et al. (2012) demonstrated that human glomerular endothelial cells possess prominent ANG II‐forming capabilities, whereas podocytes possess major ANG II‐metabolizing activity. Therefore, it was proposed that glomerular pathological conditions in which there is an imbalance in the ANG II synthetic and degradation pathways may result from selective podocyte damage. Under these circumstances, an augmented role of ANG II in glomerular disease progression may predominate, reflecting, in part, an attenuated response in the complement of podocyte peptidases (Harrison‐Bernard & Chappell, 2012). Additionally, chymase inhibition may have provided protection of the proximal tubule retrieval of albumin (Zeni, Norden, & Cancarini, 2017). It is possible that chymase inhibition blocked an ANG II degrading enzyme in control mice or that the beneficial effects of chymase inhibition on reducing albuminuria are mediated by inhibition of other pro‐albuminuric products.

Quantification of markers of glomerular matrix did not reveal a significant increase in diabetic compared to control mice infused with vehicle, which may represent a limitation of the study. Since the mice were 14 weeks of age at the time of tissue collection, renal fibrosis may not be present. It is more conventional to assess glomerular fibrosis at later time points in this animal model (Breyer et al., 2005). Diabetic mice at 16 weeks of age displayed increased mesangial expansion, fibronectin and collagen IV deposition, and desmin expansion in glomeruli compared to control mice (Kosugi et al., 2010). There may be a limitation in relying upon histological stain quantification, particularly in the absence of other quantification such as nephrin and foot process effacement.

Classically, angiotensin converting enzyme (ACE) is considered the major pathway for ANG II formation; however, evidence is mounting for an important role of chymase‐dependent ANG II formation in human tissues (Miyazaki & Takai, 2006; Miyazaki, Takai, & Jin, 2006; Takai, Jin, & Sakaguchi, 1999). Chymases are serine proteases that have chymotrypsin‐like cleavage properties for conversion of angiotensin I to ANG II at a rate 20 times greater rate than that of ACE (Reilly, Tewksbury, & Schechter, 1982; Urata, Kinoshita, & Misono, 1990) and for conversion of Ang1‐25 or Ang1‐12 to ANG II (Ferrario & Mullick, 2017). Therefore, increased chymase‐dependent ANG II formation can occur under conditions of normal or reduced ACE activity. In the current study, there was no significant difference in plasma or kidney ANG II levels between vehicle infused control and diabetic mice, as we have reported previously (Park et al., 2010). It should be noted that the ANG II levels varied greatly in the plasma and tissue samples. Such variability could mask true findings of differences.

In the db/db model, as in other diabetic mouse models, downregulation of proximal tubular ACE takes place while there is also higher ACE2 expression in kidney tubules from db/db mice (Park et al., 2010; Ye et al., 2004, 2006). Despite this configuration which would rather promote low ANG II levels, we find kidney ANG II not lower than in control mice, but rather trending to be higher which is not unexpected. Even when chymase or other non‐ACE pathways are upregulated locally within the kidney, the aforementioned configuration of ACE/ACE2 enzyme expression in diabetic mice could potentially prevent ANG II from rising in a compensatory fashion.

Elevated chymase‐dependent intracellular ANG II generation during high glucose stimulation of human mesangial cells (Cristovam et al., 2008), and mouse podocytes (Durvasula & Shankland, 2008), may provide locally elevated ANG II levels which may contribute to glomerular fibrosis. Experiments performed in isolated rat glomeruli indicated that chymase increases glomerular albumin permeability (Sharma et al., 2007), which is consistent with the findings in the current study.

Recently, Wei et al. (2010) have shown that combined chymase and ACE inhibition, relative to ACE inhibitor alone, improves left ventricular function, decreases adverse cardiac remodeling, and improves survival after myocardial infarction in hamsters. Of great interest is the observation that chronic ACE inhibition actually increases chymase expression and activity in the ischemic heart (Wei et al., 2010) highlighting the importance of targeting the chymase pathway during ACE inhibition. Since chymase contributes greatly to ANG II formation in the heart (Urata et al., 1990) we determined the heart ANG II levels in the current study. We did not find evidence for chronic chymase inhibition to reduce ANG II levels in the control or diabetic mice. Most recently, Scandiuzzi et al. (2010) have shown lower proteinuria, blood creatinine, blood urea nitrogen levels, and less severe renal histological damage in a model of glomerulonephritis in mMCP‐4 (chymase)‐deficient mice indicating an aggravating role of chymase in inflammatory kidney disease. Additionally, chronic inhibition of chymase reduced the overexpression of renal fibronectin, collagen IV, TGF‐B1 in type I diabetic rats (Fan et al., 2009). These studies support a role for a renoprotective effect of chymase inhibition.

A limitation of the current study is a lack of molecular analyses on chymase mRNA or protein changes in the kidney following treatment with the chymase inhibitor. Unfortunately, the tissue samples were used to perform ANG II peptide concentrations and histology. Future studies could help to understand the relative importance of the chymase pathway in the kidney, especially during chronic kidney disease progression, compared to the other ANG II producing pathways.

Hollenberg, Fisher, and Price (1998) estimated that in the intact human kidney at least 40% of ANGI is converted to ANG II by pathways other than ACE. He proposed that the enzyme responsible is chymase, and further predicted that the non‐ACE pathway may be substantially larger in disease states such as diabetes mellitus. Huang et al. (2003) were one of the first to identify upregulation of chymase expression in mesangial and vascular smooth muscle cells of humans with diabetic nephropathy. The enhanced renal chymase was correlated significantly with increases in blood pressure and the severity of collagen matrix deposition within the glomerulus, tubulointerstitium, and arterial walls in these patients. We have similarly reported significant increases in renal arteriolar chymase‐dependent ANG II formation in diabetic compared to control mice (Park et al., 2013), and functional evidence for enhanced chymase‐dependent renal microvascular vasoconstriction (Harrison‐Bernard et al., 2013; Park et al., 2010). We speculate that cathepsin G (Rosa et al., 2016), kallikrein, tonin, and elastase‐2 may represent alternative pathways for angiotensin‐converting enzyme‐independent ANG II formation in this study (Uehara, Miura, & Yahiro, 2013). These findings are potentially of great relevance to the treatment of patients. There may be an advantage of using chymase inhibitors compared to angiotensin receptor blockers in treating diabetic renal disease. It is possible that there is a subpopulation of DN patients that are resistant to the beneficial effects of ACE inhibition that may respond positively to chymase inhibition. We propose that combination drug therapy with chymase inhibitors and ACE inhibitors or angiotensin receptor blockers may be an effective and superior treatment for diabetic patients that fail to respond to ACE inhibitor treatment alone. These studies suggest that chymase inhibition may represent a novel therapeutic target in cardiovascular and renal disease.

## CONFLICT OF INTEREST

All authors declare that the research was conducted in the absence of any commercial or financial relationships that could be construed as a potential conflict of interest.

## AUTHOR CONTRIBUTIONS

LMHB conceived the study. LMHB, ST, and BJB designed the study. BJB, DS, and RS acquired the data. LMHB, BJB, and RS analyzed and interpreted the data. LMHB drafted the manuscript and all authors contributed to revising the document. All authors approve the final version to be published.
